# Comparative Performance of Biosorption Pretreatments for Vinasse Phytoremediation Using *Salvinia minima*

**DOI:** 10.3390/plants15193035

**Published:** 2026-10-04

**Authors:** Noelia Romina Tabernero, Silvana Chocobar-Ponce, María de Lourdes Gultemirian, Franco Suárez, Cecilia Bruno, Mariana Rosa, Carolina Prado

**Affiliations:** 1 Instituto de Bioprospección y Fisiología Vegetal (INBIOFIV), Consejo Nacional de Investigaciones Científicas y Técnicas (CONICET), Universidad Nacional de Tucumán (UNT), Tucumán 4000, Argentina; rominantab@gmail.com (N.R.T.); silvanachocobar@csnat.unt.edu.ar (S.C.-P.); cprado@csnat.unt.edu.ar (C.P.); 2 Facultad de Ciencias Naturales e Instituto Miguel Lillo, Universidad Nacional de Tucumán (UNT), Tucumán 4000, Argentina; lgultemirian@csnat.unt.edu.ar; 3 Instituto de Biodiversidad Neotropical (IBN), Consejo Nacional de Investigaciones Científicas y Técnicas (CONICET)- Universidad Nacional de Tucumán (UNT), Tucumán 4107, Argentina; 4 Cátedra de Estadística y Biometría, Departamento de Desarrollo Rural, Facultad de Ciencias Agropecuarias, Universidad Nacional de Córdoba (UNC), Unidad de Fitopatología y Modelización Agrícola (UFyMa), Instituto Nacional de Tecnología Agropecuaria (INTA), Consejo Nacional de Investigaciones Científicas y Técnicas (CONICET), Córdoba 5000, Argentina; suarezfranco@agro.unc.edu.ar (F.S.); cebruno@agro.unc.edu.ar (C.B.)

**Keywords:** batch system, fixed-bed column, phytotreatment, sugarcane vinasse

## Abstract

Sugarcane vinasse is a liquid waste generated during bioethanol production. It is characterized by high organic load, acidity, elevated salinity, and recalcitrant compounds, including soluble phenolic compounds and melanoidins, which can hinder its treatment and limit biological processes. This study evaluated batch and fixed-bed column biosorption as pretreatments coupled with phytoremediation using *Salvinia minima* at laboratory scale. System performance was evaluated based on changes in pH, electrical conductivity (EC), dissolved oxygen (DO), chemical oxygen demand (COD), true color, soluble phenolic compounds (SPCs), and the non-dialyzable melanoidin fraction. Plant physiological responses were evaluated by measuring hydrogen peroxide (H_2_O_2_) and malondialdehyde (MDA) contents. Following phytoremediation, vinasse pretreated by fixed-bed column biosorption showed greater removal of COD, SPCs, true color, and the non-dialyzable melanoidin fraction. Plants exposed to column-pretreated vinasse also showed lower H_2_O_2_ and MDA contents. In contrast, batch-pretreated vinasse adjusted to pH 6 showed an increase in the non-dialyzable melanoidin fraction after phytoremediation, suggesting structural modifications of Maillard reaction-derived compounds rather than their removal. The results indicate that the physicochemical changes occurring during biosorption improved the conditions of sugarcane vinasse for the growth of *S. minima*. Under the conditions evaluated in this study, fixed-bed column biosorption followed by phytoremediation proved to be a more suitable treatment than batch biosorption followed by phytoremediation for the integrated treatment of sugarcane vinasse.

## 1. Introduction

The expansion of the sugar–ethanol industry has increased vinasse generation, an agroindustrial wastewater produced during ethanol distillation. Due to its complex composition, vinasse management is an important issue in sugarcane-producing regions. Its direct reuse is often limited by high organic matter concentration, elevated electrical conductivity, acidic pH, and recalcitrant compounds, particularly soluble phenolic compounds and melanoidins [1,2]. Phenolic compounds have been associated with the degradation of lignocellulosic structures during sugarcane processing [3], whereas melanoidins are heterogeneous Maillard reaction products that include compounds of different molecular sizes, with the non-dialyzable fraction corresponding predominantly to higher-molecular-weight components [4]. Both groups contribute to the characteristic dark color of vinasse and show low biodegradability and high toxicity to the biota when discharged into water bodies. In addition, phenolic compounds may exert toxic effects even at low concentrations [5,6].

Vinasse also has a high nutrient content, including nitrogen, phosphorus, potassium, calcium, and magnesium, so it is frequently used for soil fertigation [7]. However, prolonged application without adequate treatment may lead to adverse outcomes, including soil salinization, contamination of water sources, increased phytotoxicity, and the release of unpleasant odors [2,7,8,9,10,11].

The environmental relevance of vinasse is further increased by the large volumes generated during bioethanol production, commonly estimated at 10–18 L of vinasse per liter of distilled alcohol [12]. To maintain the operational continuity of the alcoholic industry, it is necessary to ensure compliance with environmental regulations for the final disposal of industrial wastewater. Therefore, effective vinasse treatment is required to reduce the environmental impacts associated with its disposal [1].

Several conventional technologies have been evaluated for vinasse treatment, including physical, chemical, and biological methods [2,13,14]. However, many of these approaches present limitations related to high operational costs, energy requirements, and the generation of secondary residues such as sludge, which require additional management. These constraints may hinder their implementation, particularly at an industrial scale. Among nature-based alternatives, phytoremediation has been investigated for the treatment of agroindustrial wastewater [15]. Floating macrophytes are particularly suitable because they can tolerate contaminated conditions and contribute to the removal of nutrients, organic matter, and other pollutants [8,16,17,18,19]. In the present study, *Salvinia minima* was selected because previous studies have reported its ability to grow in wastewater and remove organic and inorganic pollutants [20,21,22,23], even in wastewater with high pollutant loads [20]. However, high salinity, elevated organic load, and the presence of recalcitrant compounds may limit plant performance and reduce phytotreatment efficiency. In this sense, biosorption using agroindustrial waste has been proposed as an alternative to improve vinasse quality before phytoremediation [9,24]. Biosorption can be applied in batch or fixed-bed column configurations, which differ in contact conditions, mass transfer, and operational mode [25,26].

Previous studies have applied biosorption and phytoremediation individually or as sequential treatments for vinasse and other agroindustrial effluents. However, the effect of biosorption pretreatment on effluent characteristics and the subsequent plant response during phytoremediation has received limited attention. Therefore, this study aimed to comparatively evaluate the performance of two distinct biosorption pretreatment systems, differing in their configuration and biosorbent components, prior to *Salvinia minima* phytoremediation of sugarcane vinasse. The treatment strategies were assessed based on changes in effluent pH, electrical conductivity, dissolved oxygen, chemical oxygen demand, true color, soluble phenolic compounds, and the non-dialyzable melanoidin fraction, together with hydrogen peroxide and malondialdehyde contents in plant tissues as indicators of oxidative stress.

## 2. Results

### 2.1. Biosorption Essay

Table 1 shows the parameters measured in raw vinasse (V_RAW) and vinasse treated using batch (VF) and fixed-bed column systems (V_COLUMN). After the batch biosorption, the VF_PH6 treatment showed lower concentrations of soluble phenolic compounds (SPCs), sucrose (SUC), and electrical conductivity (EC), as well as higher pH values, indicating better biosorbent performance. Principal component analysis (PCA) revealed that the first two components explained 80% of the system variability (Figure S1), and univariate analysis confirmed these results for VF_PH6 treatment (Figure S2). The fixed-bed column-treated vinasse showed higher pH values than the raw vinasse, while EC, SPCs, and SUC showed a marked decrease (Table 1).

**Figure 1:**
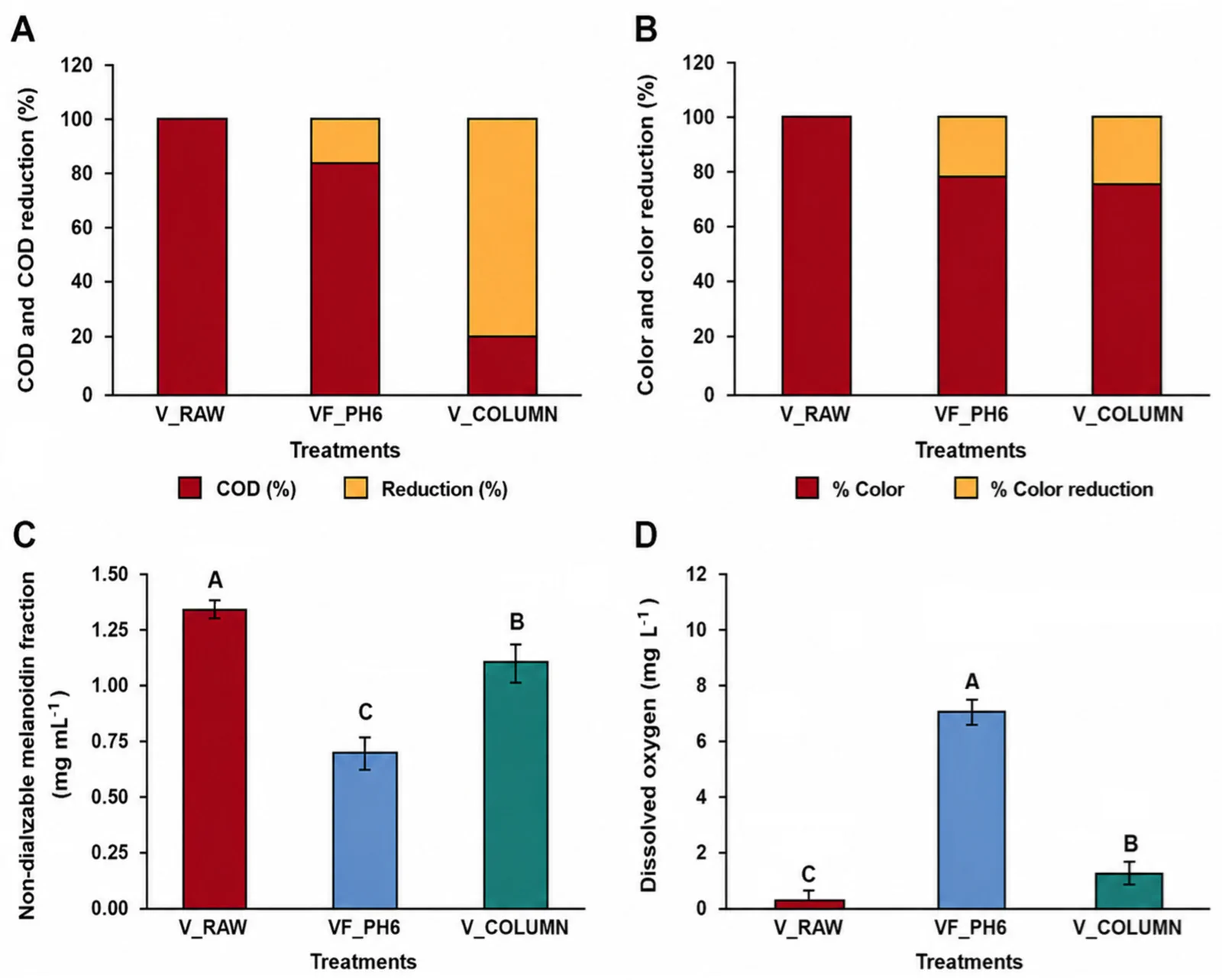
Physicochemical parameters of raw vinasse (V_RAW) and treated vinasse using batch (VF_PH6) and fixed-bed column (V_COLUMN) systems. (**A**) Chemical oxygen demand (COD) and removal efficiency (%); (**B**) true color and color removal (%); (**C**) non-dialyzable melanoidin fraction (mg mL^−1^) and (**D**) dissolved oxygen (DO). Different letters indicate significant differences among treatments (*p* ≤ 0.05).

**Figure 2:**
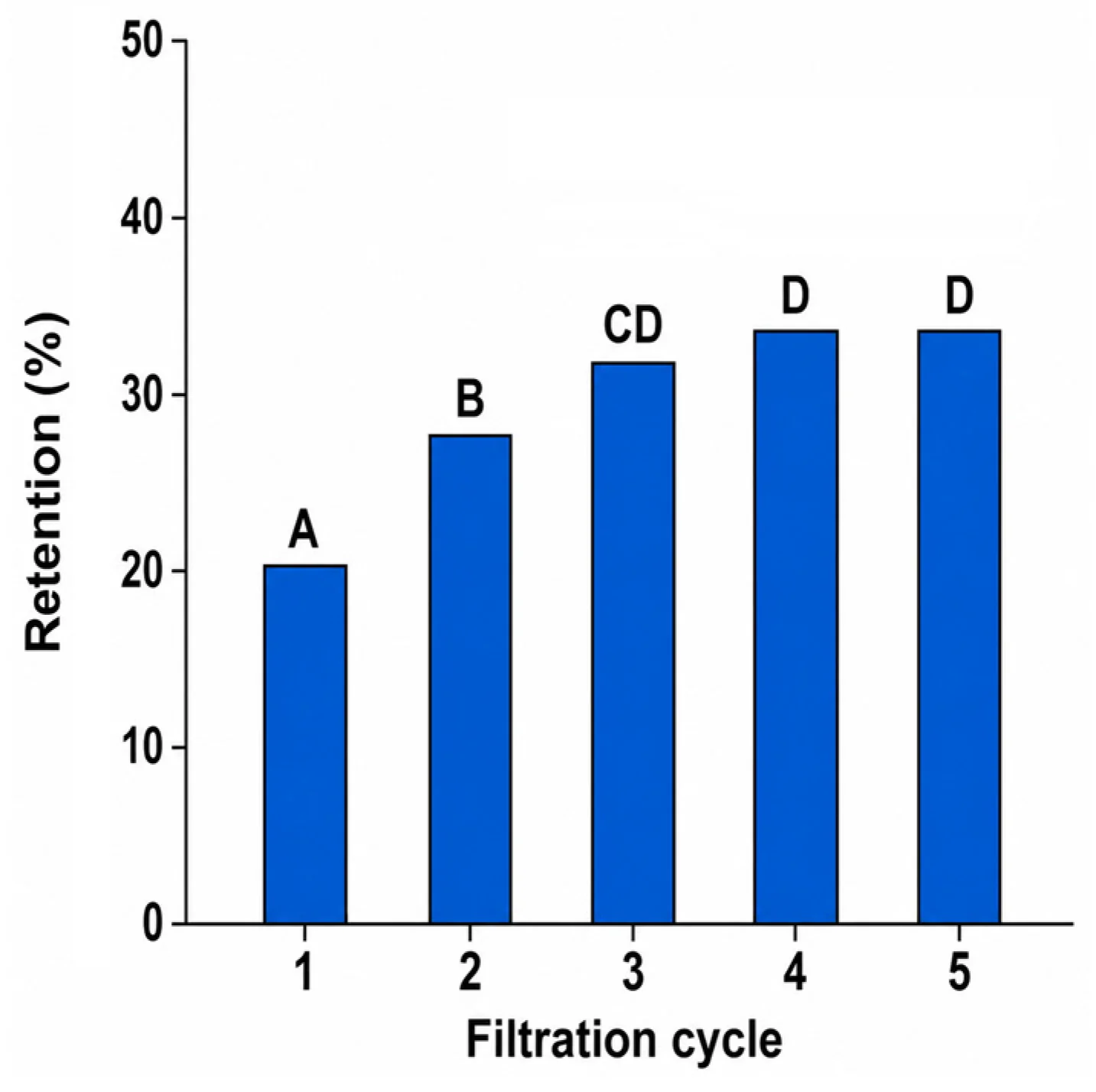
Retention percentage (%) during successive filtration cycles in a fixed-bed column system. Different letters indicate significant differences among cycles (*p* ≤ 0.05).

Based on these results, VF_PH6 and V_COLUMN treatments were selected to study chemical oxygen demand (COD), dissolved oxygen (DO), true color, and non-dialyzable melanoidin fraction, as current legislation considers these parameters for authorizing effluent discharge. The VF_PH6 treatment reduced COD by 17.68%, true color by 21.74%, and non-dialyzable melanoidin fraction by 49.13% (Figure 1A–C), while increasing DO from 0.25 ± 0.03 mg L^−1^ in the raw vinasse to 7.02 ± 0.02 mg L^−1^ (Figure 1D). The column system achieved reductions of 79.35% in COD, 23,75% in true color, and 11.23% in non-dialyzable melanoidin fraction (Figure 1A–C). DO in the column-treated effluent reached 1.95 ± 0.01 mg L^−1^ (Figure 1D). Furthermore, this system demonstrated the capacity to reduce effluent volume, achieving a retention of 33.3% of the initial volume. This retention percentage increased progressively until cycle 4, after which the recovered volume remained constant (Figure 2).

### 2.2. Phytotreatment Assays

**Figure 3:**
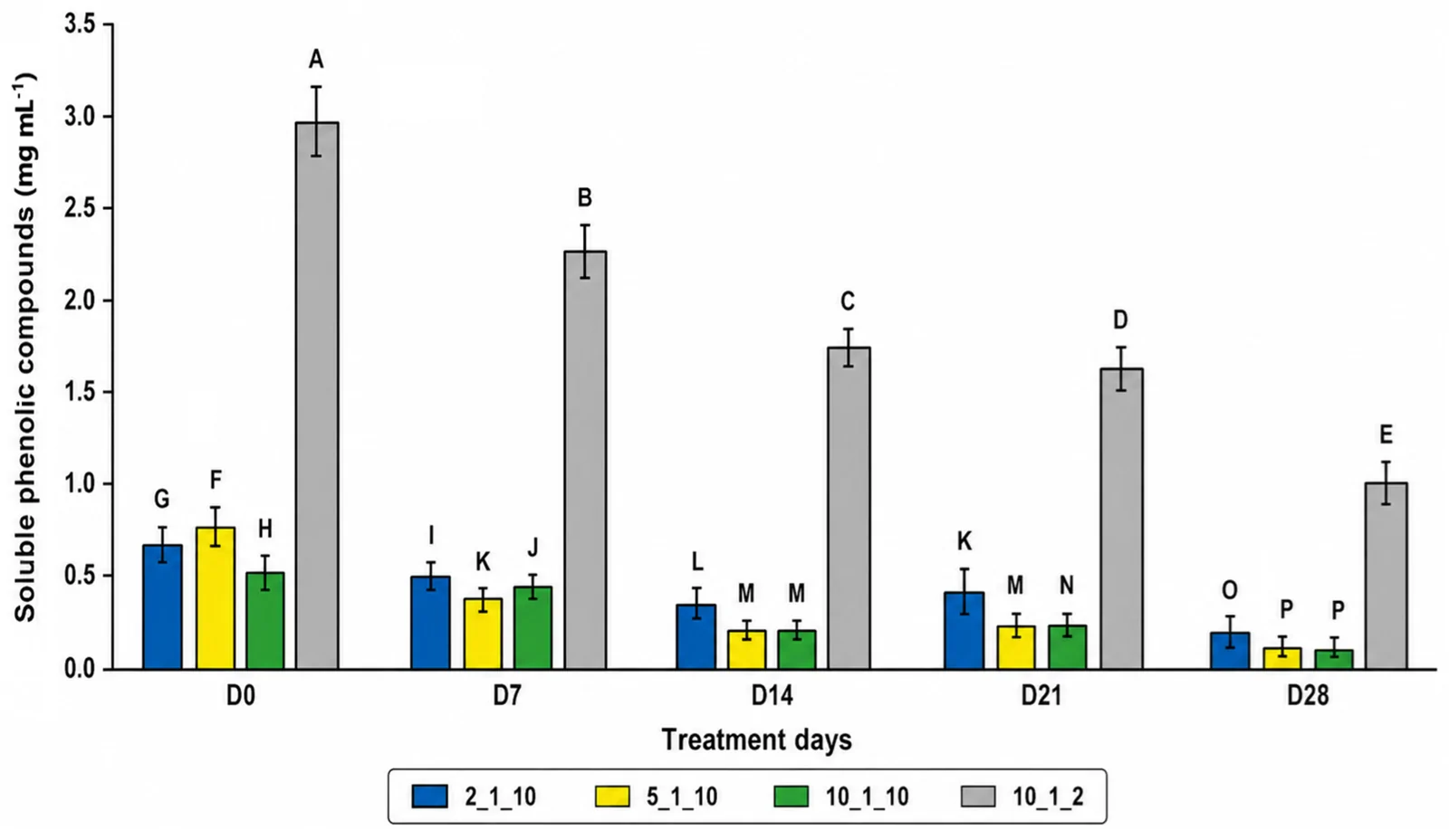
Temporal dynamics of soluble phenolic compounds (SPCs) in vinasse during optimization of the plant biomass–vinasse dilution ratio for the phytoremediation process. The treatments are represented by the following colors: blue (2_1_10), 2 g of Salvinia minima biomass with vinasse diluted 1:10; yellow (5_1_10), 5 g of biomass with vinasse diluted 1:10; green (10_1_10), 10 g of biomass with vinasse diluted 1:10; and gray (10_1_2), 10 g of biomass with vinasse diluted 1:2. Values are presented as adjusted means ± SE. Different letters indicate statistically significant differences among treatments within each sampling day (*p* ≤ 0.05).

Phytoremediation trials conducted with pure filtered vinasse proved to be highly toxic to plants, causing their death within 24 h. For this reason, it was necessary to dilute the filtered vinasse to continue with the phytoremediation process. To determine the optimal amount of plant biomass in relation to vinasse dilution, different biomass ratios and two vinasse dilutions were tested. For each ratio, SPCs, EC, and pH were measured in the vinasse, as these parameters can affect plant performance. SPCs decreased in all treatments during the experimental period, depending on the effluent dilution and the amount of biomass used (Figure 3). The 5 g–1:10 and 10 g–1:10 treatments registered the lowest values, with no statistically significant differences. The greatest decrease (84.5%) was observed in the 5 g–1:10 treatment. Regarding EC, none of the assessed proportions significantly affected its values, nor the survival of the plants. The pH increased in all treatments. No statistically significant differences were observed among the treatments with vinasse diluted 1:10 throughout the experimental period, whereas the 1:2 dilution treatment exhibited lower pH values than all other treatments. Based on these results, 5 g of plants in vinasse diluted 1:10 was chosen, as it uses less plant biomass, making it an efficient alternative under the evaluated conditions.

#### 2.2.1. Batch System Coupled with Phytoremediation

**Figure 4:**
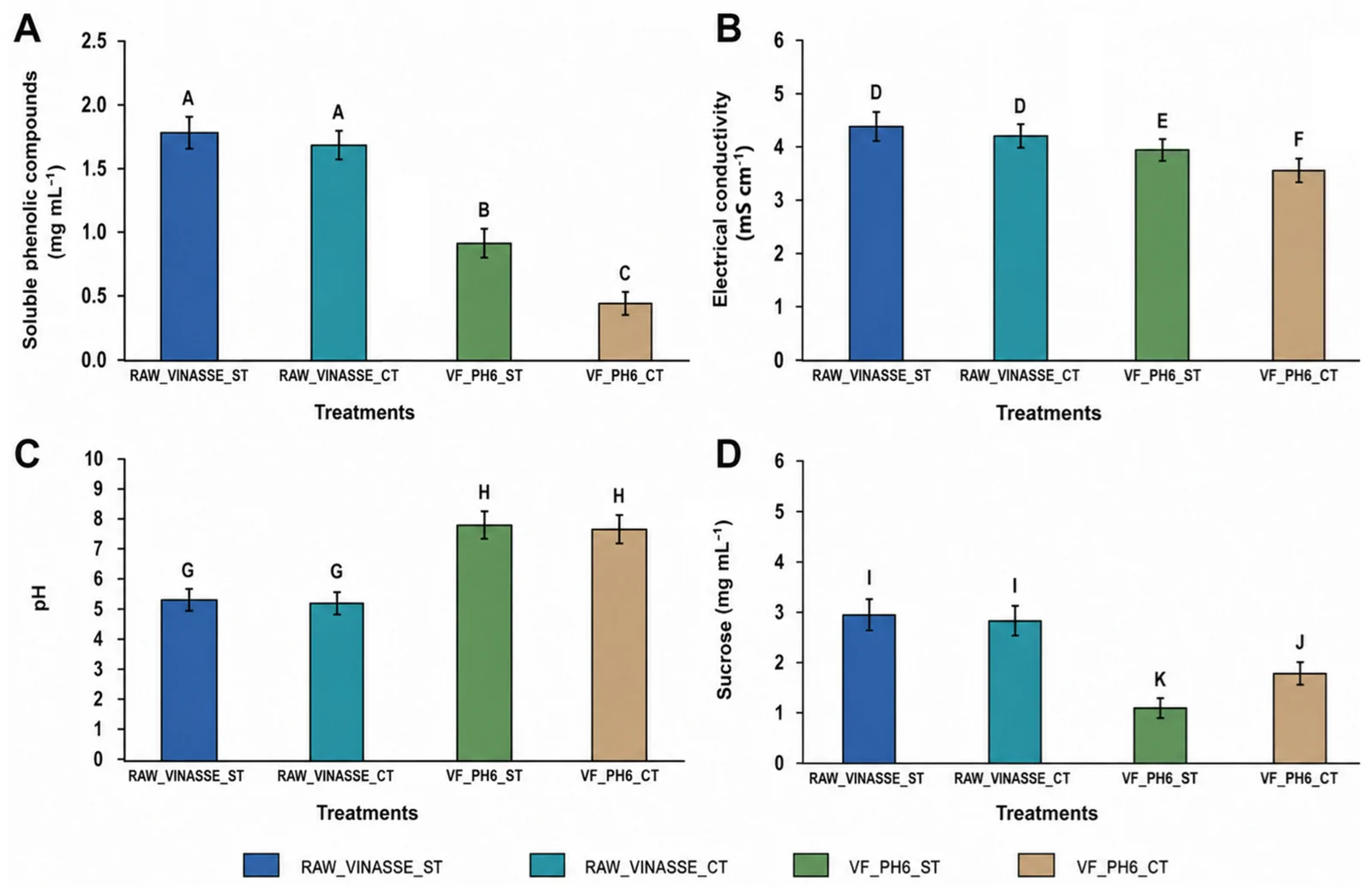
Physicochemical parameters of raw vinasse and vinasse treated by batch biosorption coupled to phytoremediation with non-pre-conditioned (ST) or pre-conditioned (CT) plants. (**A**) Soluble phenolic compounds content. (**B**) Electrical conductivity. (**C**) pH values. (**D**) Sucrose content. Bars represent adjusted means ± SE. The values shown correspond to diluted samples of both raw and treated vinasse. Different letters indicate significant differences among treatments (*p* ≤ 0.05).

For these assays, pre-conditioned (VF_PH6_CT) and non-pre-conditioned (VF_PH6_ST) plants were used, as described in Section 4.4. SPCs, SUC, pH, and EC were evaluated in both treatments and compared with the raw effluent (V_RAW) (Figure 4).

SPCs decreased across all treatments, with the greatest reduction in VF_PH6_CT, whereas V_RAW consistently maintained higher values. SUC also declined over time, reaching the lowest concentrations in VF_PH6_ST. Treatment VF_PH6_CT showed pH values close to neutrality and lower EC than V_RAW.

**Figure 5:**
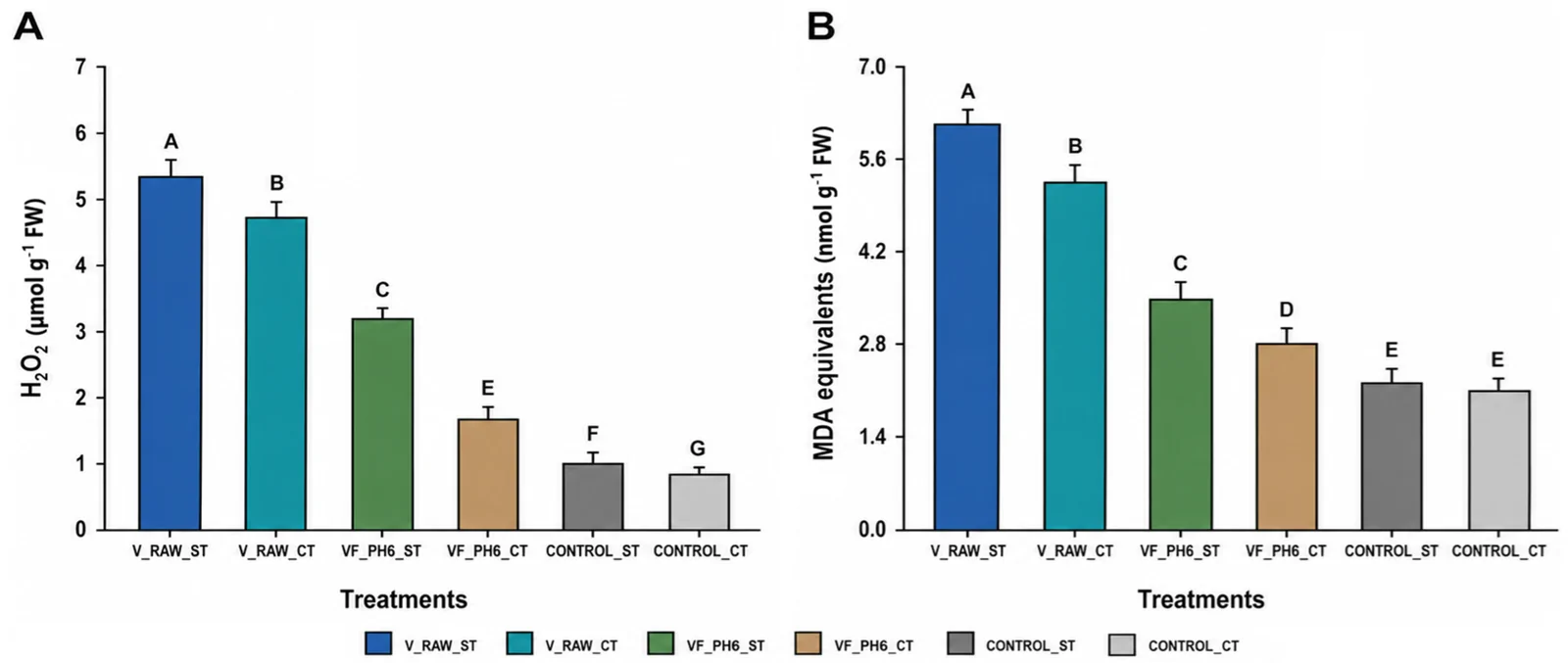
Oxidative damage indicators in *S. minima* exposed to different vinasse treatments under the batch system. (**A**) H_2_O_2_ content and (**B**) MDA content. Bars represent mean values ± SE. Different letters indicate significant differences among treatments (*p* ≤ 0.05).

In plant tissues, stress markers showed a consistent decreasing trend across treatments (Figure 5). Both H_2_O_2_ and MDA contents were higher in V_RAW and then progressively declined in treated vinasse, with lower values in VF_PH6_CT than in VF_PH6_ST, suggesting a protective effect of preconditioning. This pattern was also reflected in the principal component analysis (PCA), which associated V_RAW with higher H_2_O_2_ and MDA contents, whereas pretreated vinasse treatments were distributed at intermediate positions. VF_PH6_CT clustered near the origin, consistent with lower oxidative stress levels, while the controls clustered at the opposite end. Overall, these results indicate that phytoremediation of pretreated vinasse, particularly following preconditioning, was associated with lower oxidative stress markers in *S. minima*.

#### 2.2.2. Fixed-Bed Column System Coupled with Phytoremediation

For these assays, pre-conditioned (V_COLUMN_CT) and non-pre-conditioned (V_COLUMN_ST) plants were used, as described in Section 4.4.

**Figure 6:**
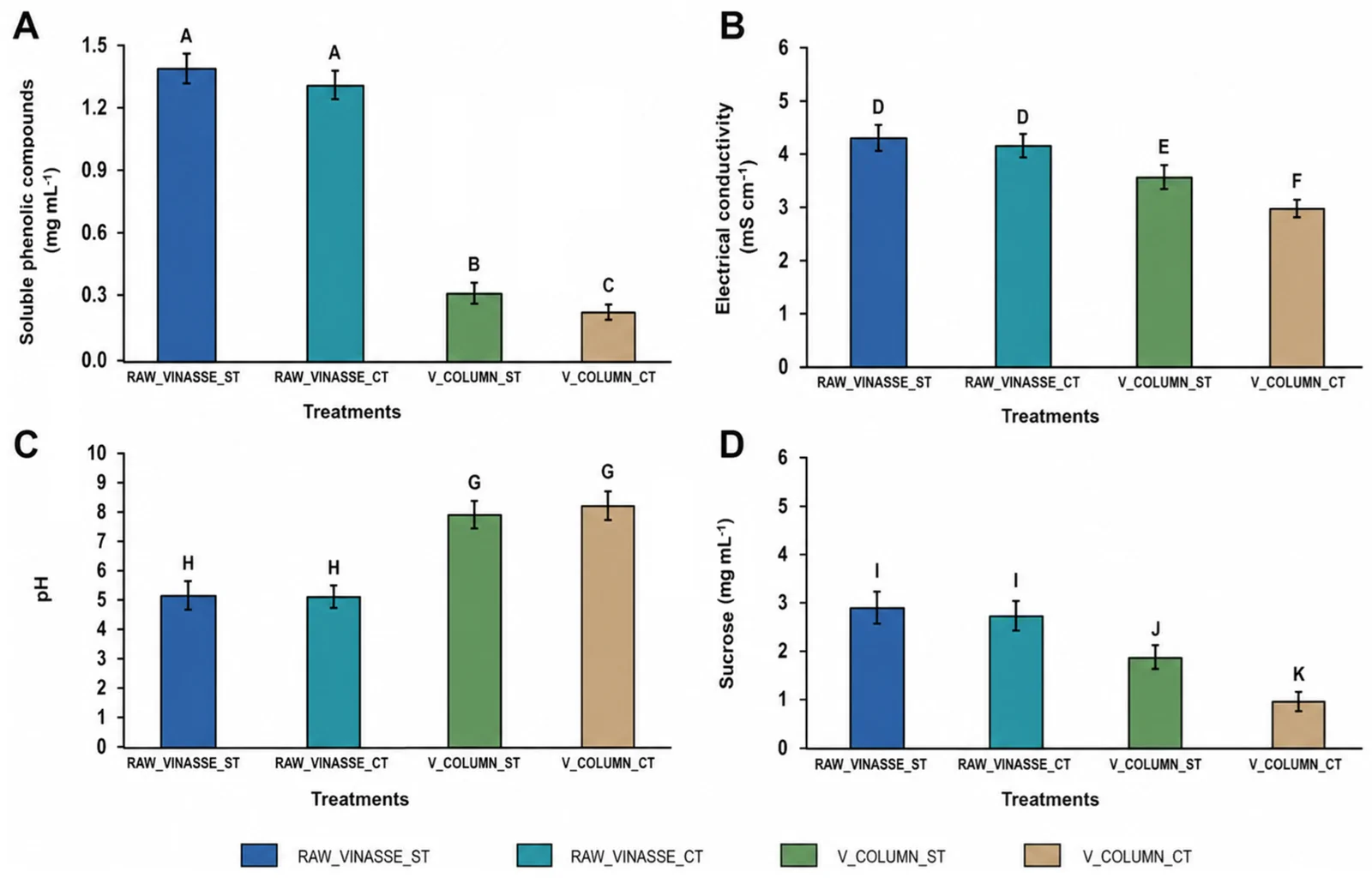
Physicochemical parameters of raw vinasse and vinasse treated by fixed-bed column biosorption coupled to phytoremediation with non-pre-conditioned (ST) or pre-conditioned (CT) plants. (**A**) Soluble phenolic compounds content. (**B**) Electrical conductivity. (**C**) pH values. (**D**) Sucrose content. The values shown correspond to diluted samples of both raw and treated vinasse. Bars represent adjusted means ± SE. Different letters indicate significant differences among treatments (*p* ≤ 0.05).

During the phytoremediation stage, the fixed-bed column-treated vinasse showed an increase in pH and a decrease in EC, SUC, and SPCs (Figure 6). This effect was more pronounced in the V_COLUMN_CT treatment, which showed better performance, with lower values of SPCs and EC and higher pH values.

**Figure 7:**
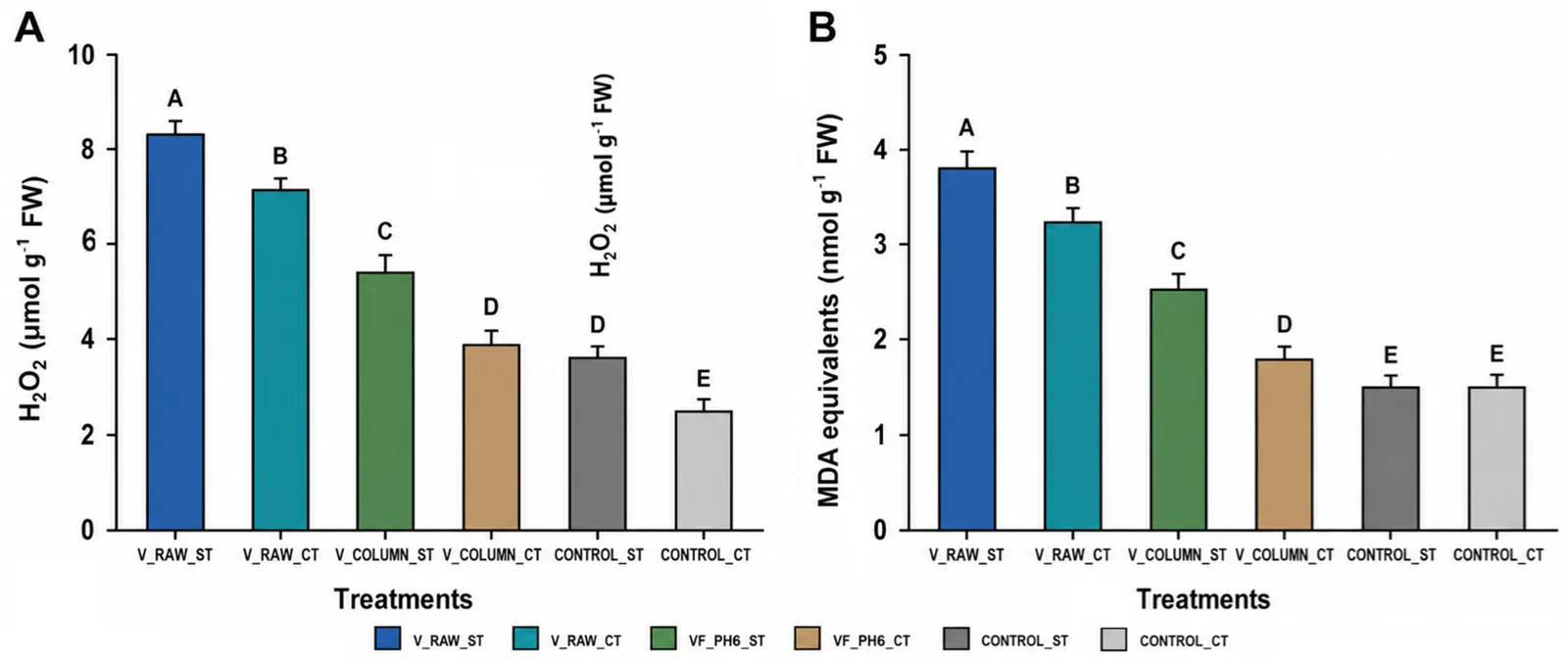
Oxidative damage indicators in *S. minima* exposed to different vinasse treatments under the fixed-bed column system. (**A**) H_2_O_2_ content and (**B**) MDA content. Bars represent mean values ± SE. Different letters indicate significant differences among treatments (*p* ≤ 0.05).

In plant tissues, oxidative stress markers showed a clear decreasing trend across treatments under the fixed-bed column system (Figure 7). Both H_2_O_2_ content and MDA were highest in V_RAW treatments and progressively declined in V_COLUMN treatments, with the lowest values observed in controls. Notably, V_COLUMN_CT exhibited lower oxidative damage than V_COLUMN_ST, suggesting a lower oxidative stress response associated with preconditioning. This pattern was also reflected in the principal component analysis (PCA), where the first two components explained 100% of the total variance (PC1: 67%; PC2: 33%). MDA and H_2_O_2_ were positively associated with PC1, while controls were positioned at the opposite end of the axis. Vinasse treatments were distributed along the positive side of PC1, with V_RAW_CT closely associated with MDA and V_COLUMN_ST with H_2_O_2_. In contrast, V_COLUMN_CT clustered near the controls, consistent with lower H_2_O_2_ and MDA levels. (Figure S4).

#### 2.2.3. Evaluation of Biosorption Pretreatments for Vinasse Phytoremediation

**Figure 8:**
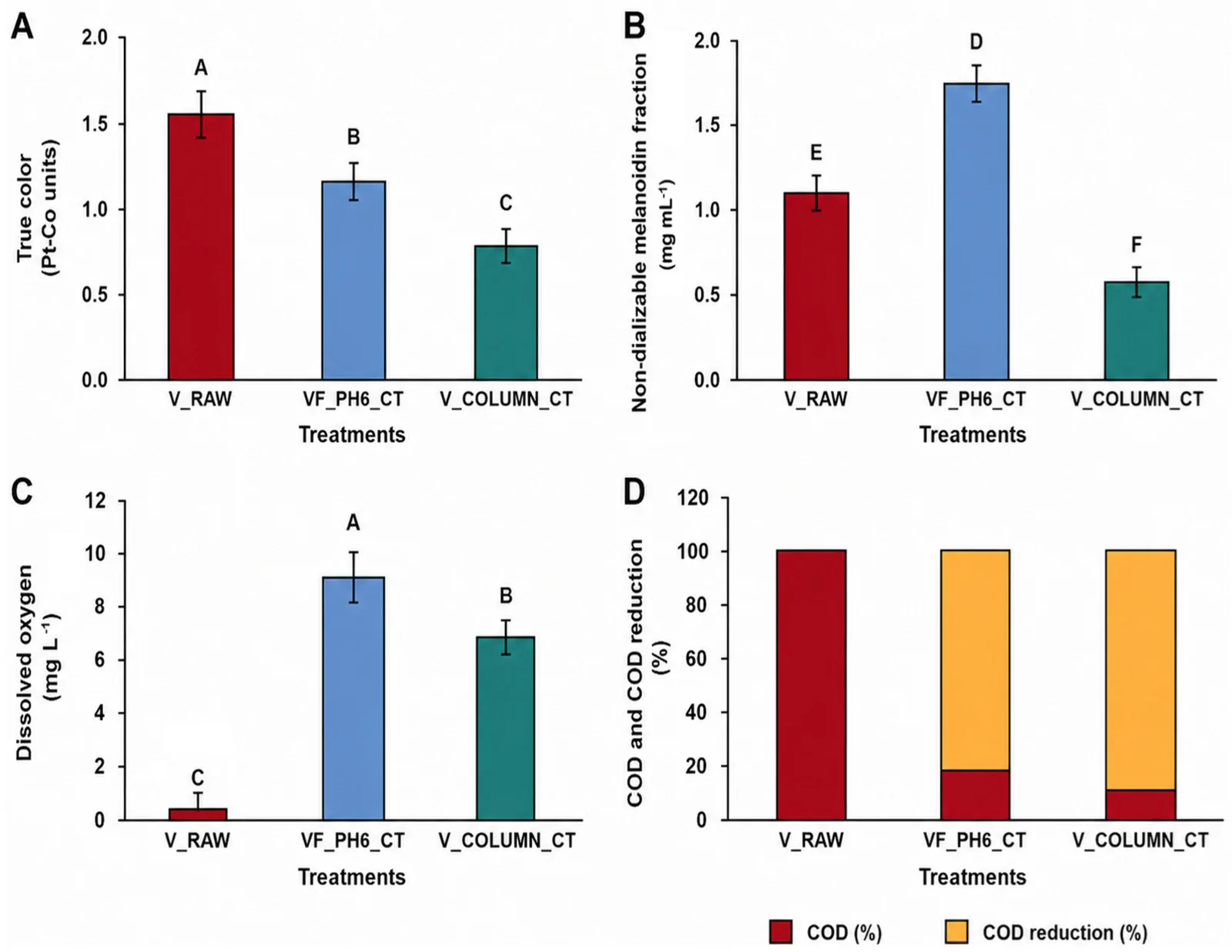
The values shown correspond to diluted samples of raw vinasse (V_RAW) and vinasse treated by biosorption systems (batch and fixed-bed column) coupled to phytoremediation with pre-conditioned *S. minima* (VF_PH6_CT and V_COLUMN_CT). (**A**) True color, (**B**) non-dialyzable melanoidin fraction, (**C**) dissolved oxygen, and (**D**) COD and COD reduction. Bars represent mean ± SE. Different letters indicate significant differences among treatments for each parameter (*p* ≤ 0.05).

Based on their overall performance, VF_PH6_CT and V_COLUMN_CT were selected for further evaluation of true color, non-dialyzable melanoidin fraction, chemical oxygen demand (COD), and dissolved oxygen (DO) (Figure 8). True color and COD decreased after both treatments, whereas changes in the non-dialyzable melanoidin fraction differed between treatments. VF_PH6_CT exhibited a higher non-dialyzable melanoidin fraction than the raw vinasse and V_COLUMN_CT. In contrast, V_COLUMN_CT showed the lowest true color, non-dialyzable melanoidin fraction, and COD values (Figure 8A,B,D). Raw vinasse exhibited a very low DO concentration (0.25 ± 0.03 mg L^−1^), which increased to 6.75 ± 0.07 mg L^−1^ in V_COLUMN_CT and 9.30 ± 0.10 mg L^−1^ in VF_PH6_CT (Figure 8C).

## 3. Discussion

The results indicate that vinasse treatment required the combination of complementary processes, as neither biosorption nor phytoremediation alone was sufficient to improve all the physicochemical parameters evaluated in this study. This finding is consistent with the complex nature of vinasse, for which no single treatment can efficiently purify the effluent [24]. The initial biosorption stage influenced the subsequent performance of the system. The greatest reductions in COD, true color, soluble phenolic compounds (SPCs), and the non-dialyzable melanoidin fraction were observed during the phytoremediation stage. Several studies have shown that aquatic macrophytes can improve the quality of pretreated effluents and have emphasized the need for preliminary conditioning stages that promote plant performance [8,19,24].

Batch experiments remain useful for laboratory-scale optimization, whereas the fixed-bed configuration provided an effluent that was more suitable for the subsequent phytoremediation stage under the conditions evaluated [25,26,27,28].

In the batch system, removal efficiency was strongly influenced by pH. This parameter regulates the surface charge and availability of functional groups of the biosorbent, as well as the speciation of contaminants in solution [29,30]. Adjustment of the effluent to pH 6 favored the removal of SPCs. In this regard, ref. [31] reported that the adsorption of phenolic compounds depends on pH, with greater removal observed around pH 6, where the non-ionized forms of many phenolic compounds predominate. Additionally, chemical activation of the biosorbent significantly improved its adsorption capacity compared to the non-activated biosorbent, according to [29,30,32]. Batch biosorption was less effective than fixed-bed column biosorption for reducing COD, true color, and the non-dialyzable melanoidin fraction, suggesting a lower removal of recalcitrant organic matter. For non-dialyzable melanoidins, a decrease in A_420_ was observed after biosorption at pH 6; however, this reduction was not maintained during phytoremediation, as VF_PH6_CT exhibited higher A_420_ values. This response cannot be interpreted as evidence of the formation of new melanoidins but is likely associated with pH-induced physicochemical modifications of pre-existing Maillard-derived compounds, which can alter their optical properties and increase absorbance at 420 nm [4,33,34]. In contrast, A_420_ continued to decrease after phytoremediation of the column-pretreated effluent, suggesting that fixed-bed column biosorption more effectively removed or transformed the color-causing compounds. In general, biosorption mechanisms include adsorption (physical or chemical), electrostatic interactions, ion exchange, chelation, complexation/coordination, covalent bonding, van der Waals forces, aggregation/precipitation (microprecipitation), and redox reactions, among others [35]. Given the complexity of vinasse, the removal of its contaminants via biosorption likely depends on interactions involving multiple mechanisms; consequently, identifying the specific mechanisms acting on each contaminant is beyond the scope of this study.

Despite this limitation, the fixed-bed column system provides practical features associated with continuous operation and the use of minimally processed waste-derived biosorbents [36,37,38]. The use of locally available agro-industrial residues may facilitate material availability and promote waste valorization within a circular economy framework by converting low-value biomass into functional biosorbent materials [35,39].

The phytoremediation stage was characterized by further reductions in SPCs, COD, EC, true color, and non-dialyzable melanoidin fraction in the effluent. Furthermore, the decrease in oxidative stress biomarkers (MDA and H_2_O_2_) in plants exposed to filtered vinasse indicated an improved physiological state, consistent with the enhanced physicochemical quality of the treated effluent [40]. Thus, phytoremediation was a key stage in the overall efficiency of the proposed system.

A further finding of this study was the association between physiological preconditioning and reduced oxidative stress in *S. minima*. These results suggest that preconditioning may improve the physiological response of *S. minima* to vinasse exposure [41]. Although their application in vinasse has been little explored, recent studies support the use of priming techniques to improve plant performance under abiotic stress conditions [41,42]. The use of non-biological primers deserves further investigation, since a change in the physiological state of the plant increases its resilience to stress conditions [41,42] and would allow the use of a smaller amount of plant biomass, making the system more efficient.

*S. minima* showed adequate performance in the remediation of filtered vinasse. This can be attributed both to the plant’s intrinsic mechanisms for absorbing, transforming, and/or degrading pollutants, and to interactions with the associated microbiota [17,19,20]. However, the results also showed that exposing the plants to raw vinasse is not viable, as it rapidly caused plant death, consistent with the findings reported by [8]. The incorporation of a prior biosorption stage was key to creating conditions compatible with plant development. These findings reinforce the need to implement combined treatments, where phytoremediation is a subsequent complementary stage [8,24,27].

Multivariate analysis provided an exploratory representation of the relationships among the physicochemical and physiological variables evaluated. The first two principal components accounted for 100% of the total variance (PC1: 77%; PC2: 23%), indicating that the selected variables adequately described the variability among treatments. The positive region of PC1 was associated with higher values of SPCs, EC, true color, and MDA, while the negative region was associated with higher pH values and a greater percentage of COD removal. In this context, V_COLUMNA_CT was located in the region associated with lower contaminant load and lower levels of oxidative stress in the plant, while VF_PH6_CT was positioned between V_RAW and V_COLUMN_CT (Figure S5). This distribution was consistent with the physicochemical and physiological patterns observed among the evaluated treatments.

## 4. Materials and Methods

### 4.1. Sample Collection

The sugarcane vinasse was provided by Ingenio La Florida-Compañía Azucarera Los Balcanes S.A., located in La Florida, Tucumán, Argentina (26°48′04″ S, 65°06′01″ W). Sampling was carried out in accordance with the protocols established by the Estación Experimental Agroindustrial Obispo Colombres (EEAOC) and the current regulations of the Secretaría de Estado de Medio Ambiente (SEMA) of Tucumán. Grab samples were homogenized to obtain a composite sample, which was stored in sterilized plastic containers at 4 °C for subsequent use.

### 4.2. Vinasse Characterization

The physicochemical properties of vinasse were characterized by measuring pH, electrical conductivity (EC), and dissolved oxygen (DO) using a WA-2017 digital meter (Lutron, Taiwan, China). Chemical oxygen demand (COD) was determined following the Standard Methods for the Examination of Water and Wastewater [43]. True color was determined spectrophotometrically at 455 nm according to the Standard Methods for the Examination of Water and Wastewater [43], using a matrix-adapted calibration procedure for vinasse. Distilled water was used as the blank, and a calibration curve was prepared from serial dilutions of untreated vinasse. Absorbance was measured using a UV–visible spectrophotometer (Hitachi U-2800A, Tokyo, Japan). Color reduction was expressed as a percentage relative to untreated vinasse.

Soluble phenolic compounds (SPCs) were quantified using the Folin–Ciocalteu method [44]. Briefly, a 50 µL aliquot was added to 0.2 mL of Folin–Ciocalteu reagent (1:1 *v*/*v* ratio) and 1.95 mL of distilled water; the mixture was allowed to stand at room temperature for 2 min. Finally, 0.8 mL of 7.5% Na2CO3 was added, and the mixture was incubated for 5 min at room temperature. Absorbance was measured at 760 nm against a reagent blank using a UV–visible spectrophotometer (Hitachi U-2800A, Japan). SPC content was calculated using a gallic acid standard curve and expressed as mg of gallic acid equivalents per gram of fresh weight (mg GAE/ml). The non-dialyzable melanoidin fraction was estimated following [45,46]. Samples were first dialyzed to remove low-molecular-weight compounds. The retained non-dialyzable fraction was then diluted at 1:10 (*v*/*v*), and its absorbance was measured at 420 nm using a UV–visible spectrophotometer (Hitachi U-2800A, Japan). Accordingly, the reported values represent the non-dialyzable melanoidin fraction rather than the total melanoidin content of the vinasse.

All analyses were performed simultaneously at room temperature throughout the experimental period.

### 4.3. Biosorption Assays

#### 4.3.1. Batch Experiments

For the batch biosorption assays, a 1:1 (*w*/*w*) mixture of fresh Citrus limon (lemon) and Arachis hypogaea (peanut) peels was used as the biosorbent. Citrus limon peel and Arachis hypogaea shells were selected as biosorbents because they are locally available agro-industrial wastes. Furthermore, both residues possess lignocellulosic matrices with functional groups suitable for the adsorption of dissolved organic compounds. The peels were washed with distilled water, oven-dried at 60 °C to constant weight, ground, and sieved to obtain a homogeneous particle size of approximately 0.2 mm. This particle size was selected based on previous studies reporting favorable adsorption performance while maintaining adequate process operability [31,47].

A portion of the biomass was sequentially treated with 0.1 M HCl and 0.1 M NaOH to obtain a chemically modified biosorbent. Chemical modification of lignocellulosic materials has been reported to enhance their adsorption properties [29,30,32]. After treatment, the modified biomass was rinsed thoroughly with distilled water until neutral pH and oven-dried at 60 °C before use.

Two identical containers were used as independent experimental units for the batch biosorption assays, using a biosorbent-to-vinasse ratio of 1:20 (*w*/*v*) under continuous agitation (150 rpm) for 24 h at room temperature. Four experimental conditions were evaluated: chemically modified biosorbent without pH adjustment (VF_pH5), chemically modified biosorbent with vinasse adjusted to pH 4 (VF_pH4), chemically modified biosorbent with vinasse adjusted to pH 6 (VF_pH6), and non-modified biosorbent without pH adjustment (VF_SHELL), which served as the control treatment.

Treatment efficiency was assessed by determining soluble phenolic compounds (SPCs) using the Folin–Ciocalteu method [44] and sucrose content according to [48]. Both parameters were measured immediately before and after the biosorption process.

#### 4.3.2. Fixed-Bed Column Biosorption Experiments

The assays were conducted using a fixed-bed column system, employing a matrix composed of different proportions and combinations of local agro-industrial waste (peanut shells, lemon peel, and pruning debris from native species), along with an inert structural support material consisting of coarse sand and gravel of heterogeneous particle size. The biosorbent material was oven-dried at 60 °C until constant weight and then hand-ground, resulting in heterogeneous particle sizes. This configuration was used to facilitate effluent passage through the packed bed. The fixed-bed column had a length of 120 cm and an internal diameter of 10 cm. The packing material occupied two-thirds of the column length and consisted of biosorbent and inert material at a 1:10 (*w*/*w*) ratio. Two identical columns were used as independent experimental units. Each column was subjected to four consecutive filtration cycles, which were considered successive operations of the same experimental unit and not independent replicates. The effluent from each column was subsequently used for the phytoremediation assays.

### 4.4. Phytoremediation Assay

Specimens of Salvinia minima were collected from an artificial lagoon located in the Horco Molle Experimental Reserve, Tucumán (26°47′32″ S, 65°18′57″ W). The plants were transported to the laboratory and carefully washed with distilled water to remove debris. The plants were then placed in containers with running water and kept under controlled conditions for 7 days to acclimate.

To determine the optimal plant biomass weight for phytoremediation, a screening was conducted using different biomass amounts and vinasse concentrations. Based on the screening results, phytoremediation was carried out in 500 mL glass beakers (8 cm in diameter and 15 cm in height) containing 400 mL of vinasse diluted 1:10 (*v*/*v*) and 5 g of *S. minima* biomass. Both treated vinasse (batch or column filtration) and raw vinasse were evaluated.

Before phytotreatment, a set of plants was preconditioned for 48 h by exposure to a commercial organic fertilizer rich in macro- and micronutrients, at the doses recommended by the manufacturer. After 48 h, the plants were removed from the preconditioning and gently washed with running water. The phytotreatment was carried out for 28 days under controlled conditions (photoperiod 12/12, temperature 24 ± 2 °C, 212 µol m^−2^ s^−1^). Plant biomass was replaced in all treatments after 7 days, except those with raw vinasse, which required additional replacements because, under these conditions, the plants died before 7 days. Plant tissue and effluent samples were taken at 7-day intervals. The physiological status of the plants was assessed by quantifying malondialdehyde content (MDA) using the thiobarbituric acid-reactive substances (TBARS) method according to Du and Bramlage [49]. Plant samples (0.5 g fresh weight) were homogenized with 3 mL of a 0.1% (*w*/*v*) trichloroacetic acid (TCA) solution and centrifuged at 12,000 *g* for 10 min. The resulting supernatants were used for MDA quantification. Briefly, 1 mL of 0.5% (*w*/*v*) thiobarbituric acid in 20% (*w*/*v*) trichloroacetic acid was added to a 1 mL aliquot of the supernatant. The resulting mixture was heated in boiling water for 25 min and rapidly cooled in an ice bath. Finally, the mixture was centrifuged at 3000 *g* for 10 min, and absorbance was measured at 440, 532, and 600 nm. MDA content was calculated using an extinction coefficient of 155 mM^−1^ cm^−1^ and expressed as nmol MDA eq./g fresh weight. H_2_O_2_ extraction and quantification were performed based on the technique proposed by Gniazdowska et al. [50], with minor modifications. First, 1 g plant tissue samples were homogenized in cold 0.1% (*w*/*v*) trichloroacetic acid (TCA) and then centrifuged at 10,000 rpm for 15 min at 4 °C. Sodium phosphate buffer (pH 7) and 10 mM potassium iodide (KI) (dissolved in sodium phosphate buffer, pH 7) were added to the recovered supernatant. The mixture was incubated for 1 h at room temperature, and the absorbance was subsequently measured at 390 nm. H_2_O_2_ concentration was determined using a standard curve prepared with a 0.892 µmol/mL H_2_O_2_ stock solution, and results were expressed as µmol H_2_O_2_/g FW. The effluent was also evaluated by measuring pH, electrical conductivity (EC), chemical oxygen demand (COD), true color (CV), soluble phenolic compound (SPC), and melanoidin (MC) content using the aforementioned techniques.

### 4.5. Statistical Analysis

All experiments were conducted using a completely randomized design. The experimental unit was the individual container for batch biosorption, the individual column for fixed-bed biosorption, and the individual vessel for the phytoremediation stage. Analytical measurements were performed in triplicate for each experimental unit and averaged before statistical analysis. These technical replicates were not treated as independent observations and therefore did not increase the sample size.

A linear mixed model (LMM) was used to evaluate pH, electrical conductivity (EC), and soluble phenolic compounds (SPCs) in relation to the plant biomass–vinasse dilution ratio during phytoremediation. pH values were log-transformed (ln pH) to meet normality assumptions, and heterogeneity of variances was modeled using a VarIdent structure. Models were fitted using restricted maximum likelihood (REML), and mean comparisons were performed using Fisher’s least significant difference (LSD) test (α = 0.05). Differences in pH (ΔpH) were analyzed using a general linear model assuming normality, independence, and homoscedasticity of errors.

The efficiency of the batch biosorption system was assessed using univariate analyses of SPCs, sucrose, EC, and pH, together with PCA as an exploratory multivariate analysis. For the fixed-bed column system, treatment differences were evaluated by analysis of variance (ANOVA). The complete treatment strategies were compared using univariate analyses of the selected final treatment parameters, complemented by PCA to explore relationships among the evaluated physicochemical and physiological variables. For all PCA, quantitative variables were standardized prior to analysis. Replicate observations were summarized by treatment using their mean values, and the resulting treatment means were used as observations in the PCA. Treatment identity was used only to identify observations in the PCA plots and was not included as a quantitative variable. The variables included in each PCA are specified in the corresponding supplementary figure legends.

All statistical analyses were performed using InfoStat software (version 2020) [51].

## 5. Conclusions

The results indicate that vinasse management requires an integrated treatment approach due to the effluent’s physicochemical complexity. Phytoremediation performance was strongly dependent on prior effluent conditioning, which supports the use of complementary processes when treating complex matrices such as vinasse. Under the conditions evaluated, the sequential combination of biosorption and *S. minima* phytoremediation improved several of the evaluated parameters, including COD, true color, and soluble phenolic compounds, while the response of the non-dialyzable melanoidin fraction depended on the biosorption pretreatment applied.

From an applied perspective, the use of agro-industrial residues as biosorbents, together with the operational simplicity of the system, supports the practical applicability of this treatment approach. Further studies should investigate biosorbent regeneration, treatment performance under variable vinasse conditions, long-term operation, and the mechanisms underlying the changes in the non-dialyzable melanoidin fraction during the sequential treatment.

## Figures and Tables

**Table 1:** Mean values ± SE of soluble phenolic compounds (SPCs), sucrose (SUC), pH, and electrical conductivity (EC) measured in vinasse treated using batch system (VF_PH6, VF_PH5, VF_PH4, VF_SHELL) and a fixed-bed column system (V_COLUMN).

TREATMENTS	PARAMETERS															
	SPC (mg/mL)				SUC (mg/mL)				pH				EC (mS/cm)			
	MEAN	±	S.E		MEAN	±	S.E		MEAN	±	S.E		MEAN	±	S.E	
V_RAW	11.18	±	0.01	A	19.11	±	0.50	A	4.93	±	0.06	C	26.7	±	0.21	A
VF_PH6	8.98	±	0.01	D	14.19	±	0.40	D	6.01	±	0.06	A	23.8	±	0.21	E
VF_PH5	9.67	±	0.02	C	15.79	±	0.03	C	4.95	±	0.06	C	25.0	±	0.21	C
VF_PH4	10.25	±	0.01	B	18.43	±	0.10	B	4.05	±	0.06	D	24.8	±	0.21	C
VF_SHELL	10.41	±	0.05	B	18.52	±	0.06	B	5.05	±	0.06	B	25.8	±	0.21	B
V_COLUMN	5.98	±	0.02	E	12.67	±	0.02	E	5.44	±	0.01	B	24.6	±	0.01	D

Notes: Different letters indicate statistically significant differences among treatments (*p* ≤ 0.05) within each variable independently. Letters are not comparable between variables. Treatments: V_RAW, untreated vinasse; VF_PH6, vinasse treated by batch biosorption with a chemically modified 1:1 (*w*/*w*) mixture of peanut and lemon peels, followed by final pH adjustment to 6; VF_PH5, same treatment without pH adjustment; VF_PH4, same treatment with final pH adjusted to 4; VF_SHELL, vinasse treated by batch biosorption with unmodified biosorbent, without pH adjustment; V_COLUMN, vinasse treated by fixed-bed column biosorption after four filtration cycles, without pH adjustment.

## Data Availability

The original contributions presented in this study are included in the article and Supplementary Material. Further inquiries can be directed to the corresponding author.

## Supplementary Materials

The following supporting information can be downloaded at: https://www.mdpi.com/article/10.3390/plants15193035/s1, Figure S1. Principal component analysis (PCA) biplot showing variability among batch biosorption treatments based on soluble phenolic compounds (SPC), sucrose (SAC), electrical conductivity (EC), and pH. Points represent treatment means, while vectors indicate the contribution and direction of each variable. PC1 and PC2 represent the first and second principal components, explaining 78.5% and 18.8% of the total variance, respectively; Figure S2. Vinasse parameters after batch biosorption. (A) Soluble phenolic compounds (SPC), (B) electrical conductivity (EC), (C) sucrose, and (D) pH. Bars represent the mean ± SE of the evaluated treatments. Different letters indicate significant differences (*p* ≤ 0.05). Figure S3. Principal component analysis (PCA) biplot showing the distribution of batch biosorption treatments coupled with phytoremediation according to malondialdehyde (MDA) and hydrogen peroxide (H_2_O_2_). Points represent treatment means for V_RAW_CT, raw vinasse with pre-conditioned *S. minima*; V_RAW_ST, raw vinasse with non-pre-conditioned *S. minima*; V_PH6_CT, batch biosorption-treated vinasse with pre-conditioned *S. minima*; V_PH6_ST, batch biosorption-treated vinasse with non-pre-conditioned *S. minima*; CONTROL_CT, control plants with pre-conditioned *S. minima*; and CONTROL_ST, control plants with non-pre-conditioned *S. minima*. Vectors indicate the contribution and direction of each oxidative stress biomarker. PC1 and PC2 represent the first and second principal components, explaining 83.4% and 16.6% of the total variance, respectively; Figure S4. Principal component analysis (PCA) biplot showing the relationship between column biosorption treatments coupled with phytoremediation and the oxidative stress biomarkers malondialdehyde (MDA) and hydrogen peroxide (H_2_O_2_). Points represent treatment means for V_RAW_CT, raw vinasse with pre-conditioned *S. minima*; V_RAW_ST, raw vinasse with non-pre-conditioned *S. minima*; V_COLUMN_CT, column biosorption-treated vinasse with pre-conditioned *S. minima*; V_COLUMN_ST, column biosorption-treated vinasse with non-pre-conditioned *S. minima*; CONTROL_CT, control plants with pre-conditioned *S. minima*; and CONTROL_ST, control plants with non-pre-conditioned *S. minima*. Vectors indicate the contribution and direction of each oxidative stress biomarker. PC1 and PC2 represent the first and second principal components, explaining 67.2% and 32.8% of the total variance, respectively; Figure S5. Principal component analysis (PCA) biplot showing the distribution of treatments based on soluble phenolic compounds (SPC), electrical conductivity (EC), pH, COD removal (%), true color, non-dialyzable melanoidin fraction (MELANOIDINS), malondialdehyde (MDA), and hydrogen peroxide (H_2_O_2_). Points represent treatment means for V_RAW, raw vinasse; V_COLUMN_CT, column biosorption-treated vinasse with pre-conditioned *S. minima*; and V_PH6_CT, batch biosorption-treated vinasse with pre-conditioned *S. minima*. Vectors indicate the contribution and direction of each variable. PC1 and PC2 represent the first and second principal components, explaining 77.2% and 22.8% of the total variance, respectively.
