# Supplementary material for: Comparative Performance of Biosorption Pretreatments for Vinasse Phytoremediation Using Salvinia minima
Source: Plants (Basel). 2026 Oct 4;15(19):3035. doi: 10.3390/plants15193035 (PMC13667176; doi:10.3390/plants15193035)
Supplement: Supplementary file 1 [file plants-15-03035-s001.zip › plants-4569166-supplementary.pdf]

Supplementary material

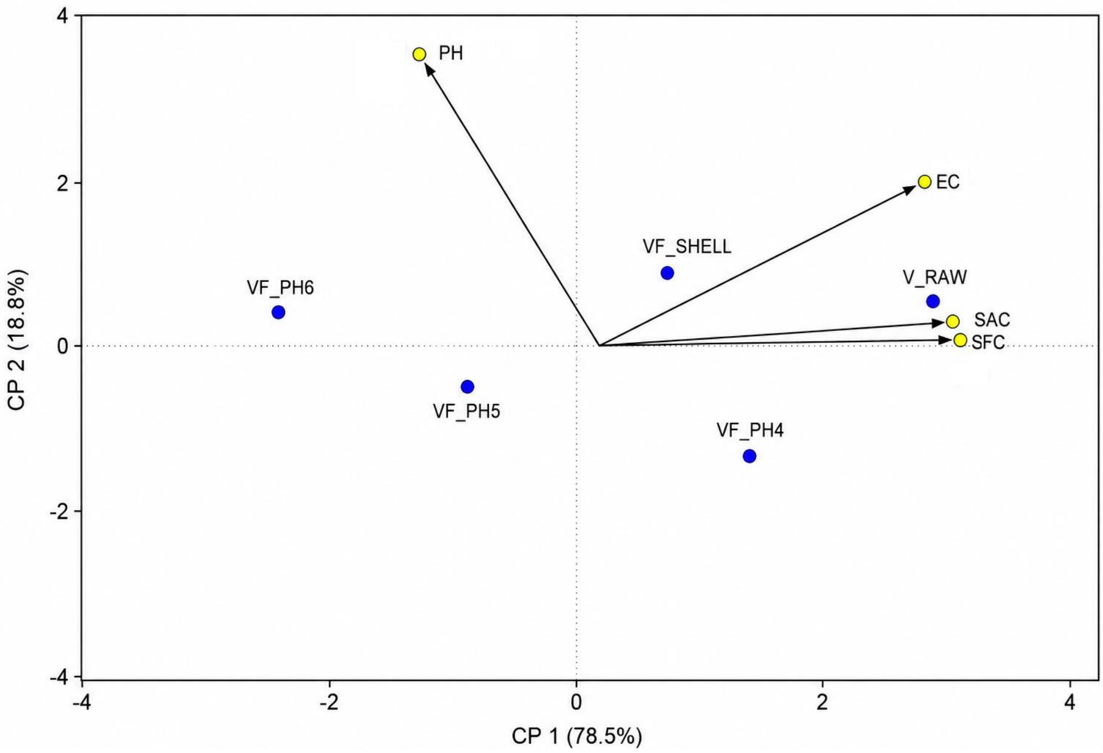

Figure S1. Principal component analysis (PCA) biplot showing variability among batch biosorption treatments based on soluble phenolic compounds (SPC), sucrose (SAC), electrical conductivity (EC), and pH. Points represent treatment means, while vectors indicate the contribution and direction of each variable. PC1 and PC2 represent the first and second principal components, explaining 78.5% and 18.8% of the total variance, respectively.

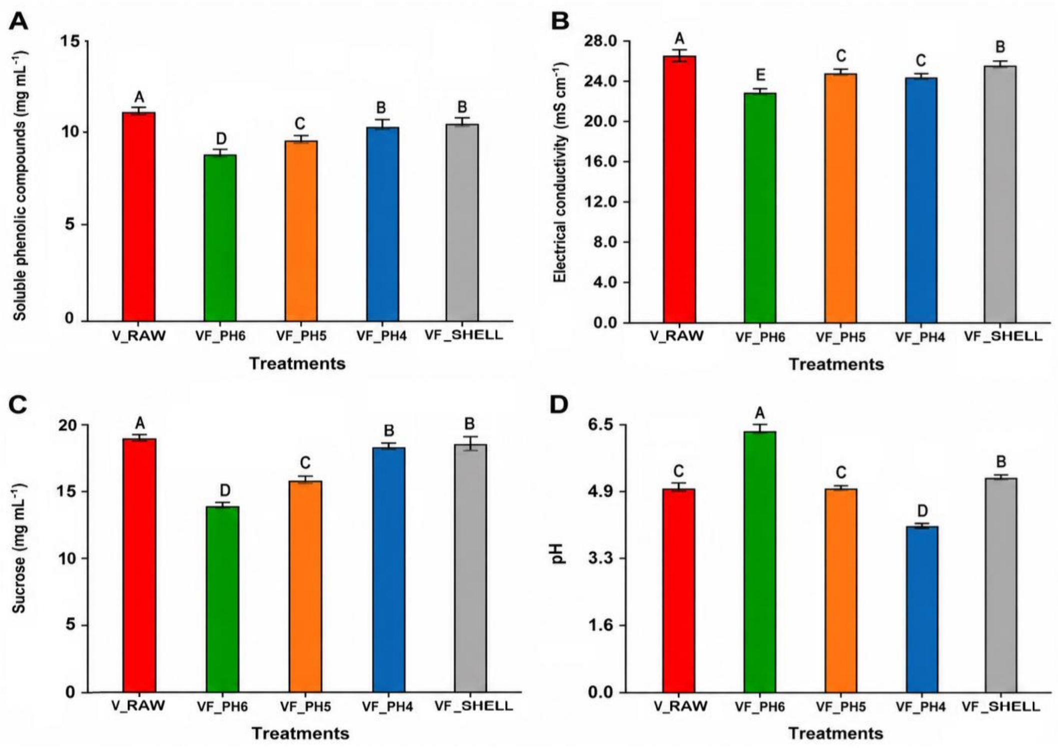

Figure S2. Vinasse parameters after batch biosorption. (A) Soluble phenolic compounds (SPC), (B) electrical conductivity (EC), (C) sucrose, and (D) pH. Bars represent the mean ± SE of the evaluated treatments. Different letters indicate significant differences (p≤0.05).

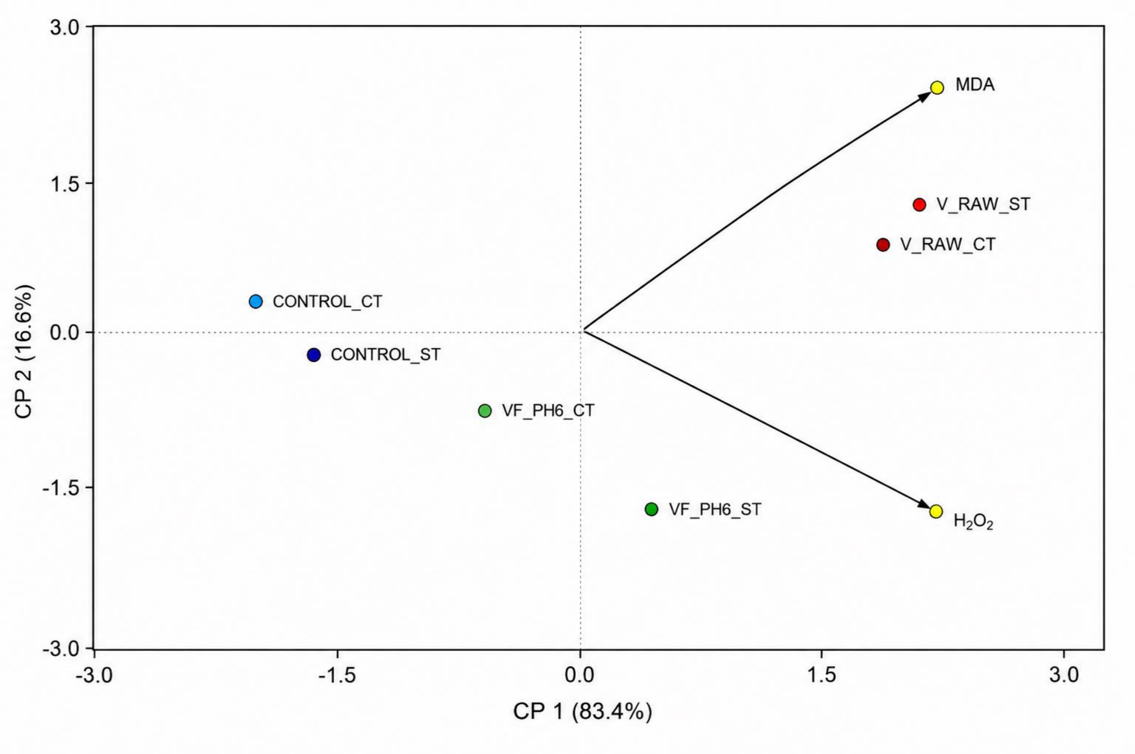

Figure S3. Principal component analysis (PCA) biplot showing the distribution of batch biosorption treatments coupled with phytoremediation according to malondialdehyde (MDA) and hydrogen peroxide (H_2O_2). Points represent treatment means for V_RAW_CT, raw vinasse with pre-conditioned *S. minima*; V_RAW_ST, raw vinasse with non-pre-conditioned *S. minima*; V_PH6_CT, batch biosorption-treated vinasse with pre-conditioned *S. minima*; V_PH6_ST, batch biosorption-treated vinasse with non-pre-conditioned *S.*

minima; CONTROL_CT, control plants with pre-conditioned *S. minima*; and CONTROL_ST, control plants with non-pre-conditioned *S. minima*. Vectors indicate the contribution and direction of each oxidative stress biomarker. PC1 and PC2 represent the first and second principal components, explaining 83.4% and 16.6% of the total variance, respectively.

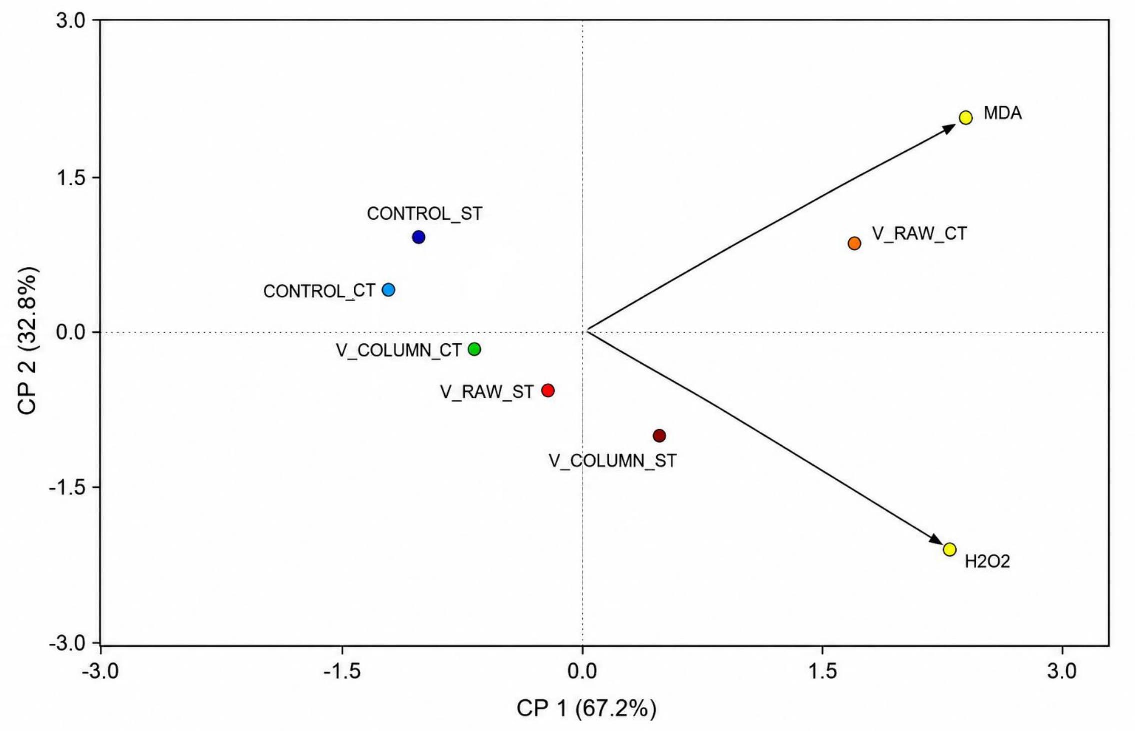

Figure S4. Principal component analysis (PCA) biplot showing the relationship between column biosorption treatments coupled with phytoremediation and the oxidative stress biomarkers malondialdehyde (MDA) and hydrogen peroxide (H_2O_2). Points represent treatment means for V_RAW_CT, raw vinasse with pre-conditioned *S. minima*; V_RAW_ST, raw vinasse with non-pre-conditioned *S. minima*; V_COLUMN_CT, column biosorption-treated vinasse with pre-conditioned *S. minima*; V_COLUMN_ST, column biosorption-treated vinasse with non-pre-conditioned *S. minima*; CONTROL_CT, control plants with pre-conditioned *S. minima*; and CONTROL_ST, control plants with non-pre-conditioned *S. minima*. Vectors indicate the contribution and direction of each oxidative stress biomarker. PC1 and PC2 represent the first and second principal components, explaining 67.2% and 32.8% of the total variance, respectively.

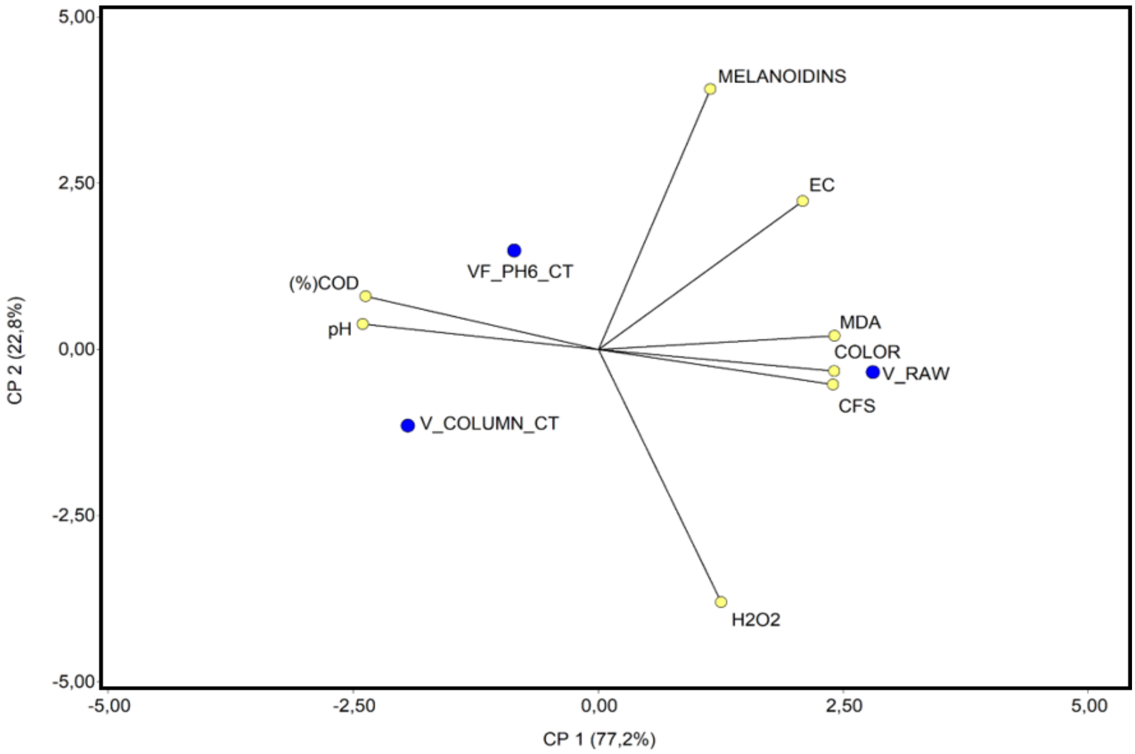

Figure S5. Principal component analysis (PCA) biplot showing the distribution of treatments based on soluble phenolic compounds (SPC), electrical conductivity (EC), pH, COD removal (%), true color, non-dialyzable melanoidin fraction (MELANOIDINS), malondialdehyde (MDA), and hydrogen peroxide (H_2O_2). Points represent treatment means for V_RAW, raw vinasse; V_COLUMN_CT, column biosorption-treated vinasse with pre-conditioned *S. minima*; and V_PH6_CT, batch biosorption-treated vinasse with pre-conditioned *S. minima*. Vectors indicate the contribution and direction of each variable. PC1 and PC2 represent the first and second principal components, explaining 77.2% and 22.8% of the total variance, respectively.
